# Commentary on the paper by Mennitti et al., Lipids in Health Disease 2014, 13:26

**DOI:** 10.1186/1476-511X-13-103

**Published:** 2014-06-24

**Authors:** Ben AR Lina

**Affiliations:** 1 TNO Triskelion, Utrechtseweg 48, P.O. Box 844, 3700 AV Zeist, Utrecht, The Netherlands

**Keywords:** Oligofructose, Offspring development

## Abstract

In a recent study by Mennitti et al., the authors concluded that oligofructose supplementation during pregnancy and lactation impairs offspring development.

However, the data reported in this article have limitations and should be interpreted with caution. A conclusion with respect to offspring development does not seem justified.

## Background

This commentary challenges the conclusion by Menitti *et al.*[1] in a recent article entitled: ‘Oligofructose supplementation during pregnancy and lactation impairs offspring development and alters the intestinal properties of 21-d-old pups’.

In this study, groups of 4 pregnant female Wistar rats were given 10% oligofructose in the diet during pregnancy and lactation. Oligofructose was incorporated at the expense of corn starch in AIN-93 based diet. On the day of delivery, litters were culled to 8 pups each.

During the 21 day lactation period, reduced body weight, body weight gain and reduced naso-anal length were reported in the offspring of dams fed the 10% oligofructose diet. In addition, retroperitoneal adipose tissue and serum concentrations of free fatty acids were reported to be decreased in pups of this group. The authors concluded that oligofructose supplementation during pregnancy and lactation impairs offspring development.

## Main text

In this commentary I would like to challenge the correctness of the conclusion in the paper by Mennitti *et al.,* 2014 that oligofructose supplementation during pregnancy and lactation impairs offspring development. In my opinion, this paper suffers from several serious limitations.

### Group size

There were only 4 dams per group, whereas, for a meaningful evaluation, international guidelines require much larger group sizes e.g. a minimum of 8 pregnant females for a Reproduction/Developmental Toxicity Screening Test (OECD 421) [2], at least 16 pregnant females per group for a Prenatal Developmental Toxicity Study (OECD 414) [3], or 20 pregnant rats for Developmental Toxicity Studies according to EPA [4] or FDA [5].

### Maternal data

Apart from the statement that ‘10% oligofructose supplementation triggered diarrhea in dams’, no information on maternal weight, feed intake or maternal toxicity was provided. This lack of information hampers evaluation of effects in pups.

### Experimental diets

The experimental diets were stated to be isocaloric (4.0 kcal/g). However, assuming an energetic value of 1.5 kcal/g for oligofructose and 4 kcal/g for corn starch, the incorporation of 10% oligofructose at the expense of corn starch results in a lower energetic value of the test diet (3.75 versus 4.0 kcal/g diet in controls). Because, moreover, no feed intake figures were presented, it is not possible to compare the energy intake in the different groups.

### Number of litters, litter size and selection of the pups

No information was provided on the selection of the pups on the day of delivery, nor on the number of selected pups per litter or on litter size. Litter size is an important determinant for the growth rate of pups. Because of this lack of information, no conclusion can be drawn with respect to the growth and size of the pups.

### Number of pups per group

Only a very limited number of pups was evaluated. According to the information provided, maximally 32 pups could be available per group. However, only 21 per group were evaluated for growth and length, while determination for adipose tissue, liver weight and serum free acid levels were conducted in 10-15 pups. The reason for selecting or rejecting pups was not reported.

## Conclusion

The data reported in this article have limitations and should be interpreted with caution. A conclusion with respect to offspring development does not seem justified.

## Competing interests

Author of this commentary is employed by TNO Triskelion. TNO Triskelion, a subsidiary of the Netherlands Organization for Applied Scientific Research, serves as independent trusted third party CRO. BENEO GmbH, Obrigheim/Pfalz, Germany commissioned TNO Triskelion to review the article by Mennitti et al. [1] and to submit the commentary for publication.
